# The impact of endometriosis on patients’ quality of sexual life

**DOI:** 10.25122/jml-2024-0262

**Published:** 2025-02

**Authors:** Mihaela Amza, Romina-Marina Sima, Ileana-Maria Conea, Tina-Ioana Bobei, Fernanda-Ecaterina Augustin, Liana Pleş

**Affiliations:** 1 Department of PhD studies, IOSUD, Carol Davila University of Medicine and Pharmacy, Bucharest, Romania; 2 Department of Obstetrics and Gynecology, Carol Davila University of Medicine and Pharmacy, Bucharest, Romania; 3 Bucur Maternity, Sf. Ioan Emergency Clinical Hospital, Bucharest, Romania

**Keywords:** endometriosis, dyspareunia, quality of life, sexual health

## Abstract

The objective of this study was to evaluate the prevalence and impact of female sexual dysfunction and sexual distress in women with endometriosis. This retrospective, analytical, observational study included patients diagnosed with ovarian endometriomas who underwent surgery to remove endometriosis lesions. The impact of endometriosis on the quality of sexual lives of patients before and after surgery was analyzed using a self-administered questionnaire consisting of 20 closed-ended questions. The study included 70 patients with endometriosis with a mean age of 32.70 ± 7.39 years. The majority of patients reported that the diagnosis of endometriosis negatively influenced their quality of sexual life (65.7%). Most patients (88.6%) experienced dyspareunia before surgery. A total of 36 patients (51.4%) stated that they had difficulty in obtaining pleasure during sexual intercourse. The intensity of dyspareunia had an important negative effect on the quality of sexual life of the patients. Following surgery, most patients (81.4%) reported improvements in their sexual quality of life, with a statistically significant reduction in pain intensity during intercourse (*P* < 0.001). These findings suggest that endometriosis may contribute to sexual avoidance and diminished pleasure. Surgical removal of endometriosis lesions significantly improved sexual quality of life, particularly by reducing dyspareunia intensity.

## INTRODUCTION

Endometriosis is one of the most common gynecological diseases, and it is present in about 10% of reproductive-age women [1]. It is characterized by the presence of endometrial-like glands and stroma outside the uterine cavity, often leading to pelvic adhesions and a spectrum of distressing symptoms, including dysmenorrhea, chronic pelvic pain, infertility, and dyspareunia [2]. Endometriosis is known to have a negative impact on patients' quality of life and has effects on mental and emotional health [3-5].

Women with endometriosis are at an increased risk of developing depression and anxiety, as their quality of life is affected not only by chronic pain but also by the emotional burden of infertility, the potential recurrence of the disease, and uncertainties related to repeated surgeries or long-term medical therapy. These factors contribute to significant sexual impairment, which may negatively affect both the psychological health of patients and their intimate relationships [6]. Several studies have explored the effects of surgical and pharmacological treatments on sexual function and relationship dynamics in women with endometriosis [7-10].

At least half of women with endometriosis may experience painful intercourse [11]. Research has shown that dyspareunia in these patients is often linked to a variety of sexual dysfunctions, including low sexual desire, difficulties with lubrication, impaired arousal, and orgasmic disorders [12-14]. Furthermore, the anticipation and fear of pain from repeated distressing sexual experiences can act as potent inhibitors of the sexual response cycle, further exacerbating sexual dysfunction [15].

The objective of this study was to investigate the prevalence and impact of female sexual dysfunction and sexual distress in women with endometriosis. Additionally, we assessed the effect of surgical removal of endometriosis lesions on patients' quality of sexual life.

## MATERIAL AND METHODS

This retrospective, analytical, and observational study was conducted between April and June 2023. The study included patients admitted to our hospital between January 2018 and March 2023 who were diagnosed with ovarian endometriomas and underwent surgery to excise endometriosis lesions. Patient selection was based on the ENZIAN classification system [16]. Only patients with ovarian endometriosis were included in this study (the sum of the diameters of all endometriomas at least equal to 3 cm — according to the ENZIAN classification O2 or O3). Superficial peritoneal endometriotic lesions (ENZIAN P0, P1, P2, or P3) could be present. Patients with deep infiltrating endometriosis were not included in this study. Additional exclusion criteria were irregular periods or symptoms associated with perimenopause, such as sleep disturbances, hot flashes, night sweats, or irregular/skipped periods.

Patients were contacted and asked to complete a self-administered questionnaire consisting of twenty closed-ended questions. All participants included in the study answered the questionnaire at least 3 months after surgery. We collected data about symptoms associated with endometriosis but also about their sexual life. The impact of symptoms caused by endometriosis on the quality of sexual life was assessed. We evaluated the changes that occurred in terms of sexual life after the surgical intervention. A detailed analysis was performed regarding dyspareunia. Pain intensity during sexual intercourse was assessed using a visual analog scale from 1 to 10, where 1 represents mild pain and 10 represents unbearable pain.

All collected data were entered into a custom database and analyzed using SPSS Statistics version 23 (IBM Corp., Armonk, NY, USA). We used descriptive statistical methods to calculate the mean, standard deviation, and frequencies. To assess statistically significant relationships between parameters, we used the independent sample *t*-test, paired samples *t*-test, and Fisher's exact test. A *P* value < 0.05 was considered statistically significant.

## RESULTS

A total of 70 patients diagnosed with ovarian endometriomas who underwent surgical excision of endometriotic lesions were included in this study. The participants were between 21 and 49 years old at the surgery, with a mean age of 32.70 ± 7.39 years. Superficial peritoneal endometriotic lesions were identified in 15 patients (21.4%). In all cases, ovarian cystectomy was performed. Following surgery, all participants received combined oral contraceptives for 3 months. All participants reported experiencing pelvic pain. In addition, other symptoms such as dysmenorrhea (72.9%), infertility (15.7%), metrorrhagia (7.1%), dysuria (1.4%), hematuria (1.4%) and pollakiuria (1 .4%) were also present. The majority of patients stated that the diagnosis of endometriosis negatively influenced their quality of sexual life (65.7%). A number of 30 participants (42.9%) reported that they avoid sexual intercourse due to the symptoms caused by endometriosis.

**Figure 1:**
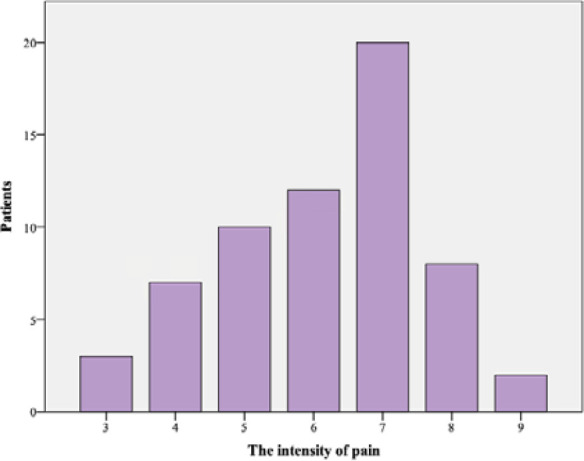
The intensity of dyspareunia before surgery

Before surgery, dyspareunia was present in 62 patients (88.6%), with a mean pain intensity score of 6.15 ± 1.48 on a visual analog scale (VAS) (Figure 1). Despite this, 85.7% of participants reported experiencing pleasure during intercourse. Out of these, 36 patients (51.4%) stated that they had difficulty achieving sexual pleasure.

Independent sample *t*-test showed that the mean pain intensity during sexual intercourse was significantly higher among patients who considered that the diagnosis of endometriosis negatively influenced the quality of their sexual life (*P* < 0.001) and who avoided sexual intercourse due to symptoms caused by endometriosis (*P* = 0.02). Furthermore, dyspareunia was significantly more intense in patients who experienced difficulty or an inability to achieve pleasure during intercourse (*P* = 0.01).

We used Fisher's exact test and concluded that there was no statistically significant association between experiencing sexual pleasure during intercourse and avoiding intercourse due to symptoms caused by endometriosis (*P* = 0.08). However, a significant association was observed between the perception that an endometriosis diagnosis negatively impacts sexual quality of life and the ability to experience pleasure during intercourse (*P* = 0.02).

**Figure 2:**
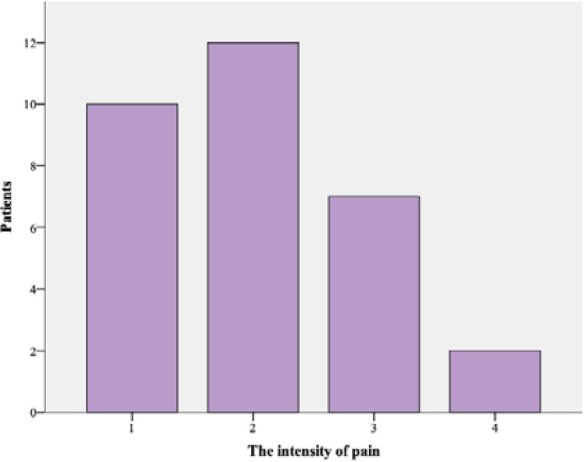
The intensity of dyspareunia after surgery

Participants resumed sexual activity between 4 and 52 weeks after surgery (11.01 ± 6.8 weeks), with 31 patients (44.3%) resuming intercourse between 8 and 9 weeks postoperatively. Most patients (81.4%) considered that there were changes in the quality of sexual life after surgery. Out of the total, 42 participants (60%) considered that pain during sexual intercourse improved, and only 15 participants (21.4%) stated that dyspareunia disappeared. Thirty-one participants (44.3%) reported they continued to experience dyspareunia after surgery. Patients evaluated the intensity of pain during sexual intercourse after surgery using a visual analog scale (Figure 2). The mean VAS score for dyspareunia decreased significantly to 2.03 ± 0.91 (Figure 2). Out of the total, 66 participants (94.3%) reported pleasure during sexual intercourse, and 58 participants (82.9%) did not have difficulty to get pleasure. Additionally, 45 patients (64.3%) reported an increased frequency of intercourse, while 20 patients (28.6%) considered that there was no change in the frequency. The majority (92.9%) stated that their partners did not experience discomfort during intercourse.

We compared the intensities of dyspareunia in 31 patients who reported pain during sexual intercourse after surgery using the paired samples *t*-test. The mean pain intensity was significantly lower after surgery (*P* < 0.001).

## DISCUSSION

Sexual health is a very important aspect of women's lives, and it is frequently influenced by gynecological conditions [17]. In a study by De Graaff *et al*., which included 931 patients with endometriosis, chronic pelvic pain was reported in 60% of participants, dysmenorrhea in 57%, and dyspareunia in 47%. The symptoms associated with endometriosis affect the physical and mental health of patients [18]. Endometriosis can be associated with a high level of stress and psychiatric disorders such as depression or anxiety [19]. An important percentage of the patients included in our study (65.7%) stated that endometriosis negatively affected their sexual lives. Endometriosis has been shown to significantly impact couple dynamics. A fear of separation was found due to the decrease in the frequency of sexual intercourse. Women diagnosed with endometriosis may develop a feeling of guilt towards their partner. Additionally, the condition can negatively affect self-esteem, leading to diminished self-respect and reduced feelings of femininity. Communication within couples, particularly regarding sexual health, often deteriorates following an endometriosis diagnosis [20]. The association between endometriosis and infertility is well known [21]. In addition, the sexual life of a couple is negatively impacted by infertility problems. Sexual dysfunction was frequently reported among couples with infertility (48%-58% among men and 43%-90% among women) [22]. In the case of patients with endometriosis, sexual life can be affected by infertility problems, not only by the painful symptoms associated with endometriosis. Thus, it is difficult to identify how much infertility or endometriosis separately affects the sexual life of patients [23].

Sexual well-being is essential for relationship satisfaction and overall quality of life. Several factors, including age, educational level, and chronic diseases, have been identified as contributors to variations in sexual function [24,25].

In our study, we observed that 92.9% of patients' partners did not report discomfort during sexual intercourse, suggesting that partner perception plays a critical role in sexual health outcomes. Hämmerli *et al*. reported that the majority of partners observed changes in sexual activity after the diagnosis of endometriosis. Partners of patients with endometriosis were less satisfied with their sexual lives, and in most cases, the frequency of sexual intercourse was reduced [26]. Despite these challenges, some partners perceived women with endometriosis as resilient individuals and developed a sense of admiration for their ability to cope with the condition [27].

In terms of treatment, extensive surgery for deep endometriosis is a feasible method but is associated with a number of complications. Many studies have reported an improvement in dyspareunia after surgery [28,29]. A review that evaluated 17 articles concluded that laparoscopy used in the treatment of endometriosis can improve the quality of sexual life [30]. In our study, a statistically significant decrease in the intensity of pain during sexual intercourse was observed.

Dubuisson *et al*. evaluated the quality of sexual life in patients diagnosed with deep endometriosis one year after surgical intervention and reported significant improvements. Their findings indicated a more relaxed mood among partners, an increased frequency of sexual intercourse, and greater satisfaction with overall sexual experience, including orgasmic function [31]. Similarly, Malekmaleki *et al*. observed an improvement in the Sexual Quality of Life (SQOL) score six months after laparoscopic excision of endometriotic lesions [32]. Dior *et al*., which included 149 patients diagnosed with deep endometriosis who underwent surgical treatment, found no significant improvement in sexual life at 6 weeks postoperatively. However, by 6 and 12 months, patients reported significant improvements in sexual desire, arousal, and pain reduction [33].

Sexual life represents an important part of the patient's quality of life, being a determinant of well-being. A series of social, emotional, and cultural factors are also involved in this relationship. It is necessary to understand what are the pathologies that affect the quality of sexual life and how they can be influenced by treatment.

## CONCLUSION

Endometriosis can negatively affect the sexual life of patients. Dyspareunia may be associated with avoidance of sexual intercourse or difficulty in achieving sexual pleasure. The intensity of dyspareunia is associated with the negative impact of endometriosis on the quality of sexual life. Following the surgical excision of endometriotic lesions, patients reported improvements in sexual well-being, including a reduction in dyspareunia intensity.

## Data Availability

Further data is available from the corresponding author upon reasonable request.
